# Experimental posterolateral spinal fusion with beta tricalcium phosphate ceramic and bone marrow aspirate composite graft

**DOI:** 10.4103/0019-5413.67118

**Published:** 2010

**Authors:** Ankit Gupta, Vijendra Chauhan, Neena Chauhan, Sansar Sharma, Rajesh Maheshwari, Atul Agarwal

**Affiliations:** Departments of Orthopaedic Surgery and Pathology, Himalayan Institute of Medical Sciences, Swami Ram Nagar, Jollygrant, Doiwala, Dehradun- Uttarakhand, India

**Keywords:** Beta tricalcium phosphate, bone marrow aspirate, posterolateral spinal fusion

## Abstract

**Background:**

Beta tricalcium phosphate is commonly used in metaphyseal defects but its use in posterolateral spinal fusion remains controversial. There are very few published animal studies in which use of beta tricalcium phosphate has been evaluated in the posterolateral lumbar arthrodesis model. Hence we conducted a study to evaluate the potential of composite graft of beta tricalcium phosphate and bone marrow aspirate in comparison to autologous bone graft, when used for posterolateral spinal fusion.

**Materials and Methods:**

Single level posterolateral lumbar fusion was performed in 40 adult male Indian rabbits, which were assigned randomly into one of the four groups based on graft materials implanted; a) 3 gm beta tricalcium phosphate plus 3 ml bone marrow aspirate (Group I); b) 3 ml bone marrow aspirate alone (Group II); c) 3 gm beta tricalcium phosphate (Group III) and d) 3 gm autologous bone graft (Group IV). Each group had 10 rabbits. Half of the rabbits were sacrificed by injecting Phenobarbitone intraperitoneally after eight weeks and the remaining after 24 weeks, and were evaluated for fusion by X-rays, computed tomography (CT) scans, manual palpation test and histology.

**Results:**

Beta tricalcium phosphate used with bone marrow aspirate produced best results when compared to other groups (*P* =.0001). When beta tricalcium phosphate was used alone, fusion rates were better as compared to fusion achieved with autologous iliac crest bone graft though statistically not significant (*P* =0.07). Autologous bone graft showed signs of new bone formation. However, the rate of new bone formation was comparatively slow.

**Conclusion:**

Composite graft of beta tricalcium phosphate and bone marrow aspirate can be used as an alternative to autologous iliac crest bone graft.

## INTRODUCTION

The use of bone graft is a common practice during spinal surgeries. Autologous graft from iliac crest is recognized as the most successful bone graft material and is presently the “gold standard” against which all other graft materials are compared.^1^ However, donor site morbidity, increased blood loss and operative time and risks of nerve injury occur in relatively high number of cases.^2^,^3^ Additionally, pseudoarthrosis with the use of autologous bone graft has been reported to occur in about 30% of the cases.^4^,^5^ The quantity of bone available to harvest may not be sufficient for long, multilevel fusion or in patients with previous graft harvests. In order to overcome these problems, it is desirable to develop graft materials which have osteoconductivity as well as osteoinductivity equal to that of natural bone; and which can take the place of autologous bone graft.^1^ A number of ceramic bone graft substitutes have received attention as alternative to autologous bone graft, chief being hydroxyapatite [Ca_10_ (PO_4_)_6_(OH)_2_] and tricalcium phosphate [Ca_3_ (PO_4_)_2_], which are biocompatible and osteoconductive materials that offer a chemical environment and a surface conducive to new bone formation.^6^–^9^ These are brittle materials and have low fracture resistance. They lack osteogenic and osteoinductive properties present in an autologous bone graft. In order to provide these properties various osteogenic and osteoinductive material can be added to make them an ideal graft substitute.^10^ Commercially available hydroxyapatite is resorbed very slowly, if at all, under normal physiological conditions.^10^ A fully nonresorbable graft material may hinder remodeling, cause strength deficiency of new bone and leave permanent stress risers in the fusion mass.^11^ An appropriate ceramic porous beta tricalcium phosphate is biodegradable material that can be replaced by bone tissue.^12^,^13^ The acceptance of these substitutes by host tissues is determined by two important features: pore diameter and the porosity or interconnectivity. Minimum pore size of 100 *μ*m is optimal for bone ingrowth and a pore size of more than 200 *μ*m facilitates development of mature osteon.^14^ Bone marrow harvested by aspiration contains osteoblastic progenitors with other bone marrow-derived cells that are rich in cytokines. It also provides a degradable biologic matrix of fibrin, which can revascularize rapidly.^15^,^16^ We selected porous beta tricalcium phosphate as a scaffold and combined it with bone marrow aspirate for bone regeneration. We conducted a study to evaluate the potential of composite graft of beta tricalcium phosphate and bone marrow aspirate in comparison to autologous bone graft when used for posterolateral spinal fusion.

## MATERIALS AND METHODS

Forty adult male Indian rabbits (n = 40), each weighing 2.5 – 3.5 kg and approximately one-year-old were obtained from a rabbitry and were operated upon after obtaining permission from the Institutional Animal Ethical Committee (IAEC). The rabbits were housed, one per cage, and given nutritive diet. They were inspected daily for general health and neurologic conditions. The animals were randomly allocated to receive one of the following graft materials: a) 3 gm beta tricalcium phosphate plus 3 ml bone marrow aspirate (Group I); b) 3 ml bone marrow aspirate alone (Group II); c) 3 gm beta tricalcium phosphate (Group III) and d) 3 gm autologous bone graft (Group IV). Each group consisted of 10 rabbits (n=10). A total 3 ml of bone marrow was aspirated from one or both the iliac crests in Group I and Group II using bone marrow aspiration needle. 3 gm of autologous bone graft was harvested from one or both iliac crests in Group IV after exposing the iliac crest.

Porous beta tricalcium phosphate ceramic (chronOS, Synthes GmbH, Switzerland) with porosity of 60% and pore width of 100 – 500 *μ*m was used in group I and III. The surgical procedure was the same in all the animals.

### Operative procedure

Under general anesthesia (40 mg/kg of intramuscular ketamine and 0.2 mg/kg diazepam) and after preparation of the part, a dorsal midline skin incision was made and spinous processes were identified. Subperiostealy, muscles were stripped and spinous processes, lamina and transverse processes of the lower lumbar vertebrae were exposed on both sides. With a small rongeur the transverse processes, spinous processes, and laminae were decorticated and half of prepared graft material were positioned on the graft bed on each side as follows: in Group I, 3 gm of beta tricalcium phosphate was soaked in 3 ml of bone marrow aspirate and then half of the total substance thus formed was grafted on each side over the prepared decorticated bed; in Group II, 3 ml of bone marrow was aspirated as described and was injected equally over the prepared decorticated area; in Group III, 3 gm of beta tricalcium phosphate was soaked in saline and then half of the total substance thus formed was grafted on each side; in Group IV, 3 gm of autologous bone graft was harvested and then half of the total substance thus obtained was grafted on each side. The wound was closed in layers after achieving hemostasis.

Animals were kept fasting for three to four hours postoperatively. Injection Cefotaxime 20 mg/kg body weight, was given intramuscularly, everyday, for three days.

### Follow-up

Half the rabbits (n=20, five from each group), were sacrificed by injecting Phenobarbitone intraperitoneally after eight weeks, the remaining half (n=20, five from each group) after 24 weeks and all were evaluated for fusion by X-rays, CT scans, manual palpation test and histology to increase the accuracy of assessing the fusion.

### Assessment of fusion

#### Radiological analysis

**Figure 1(a,b):**
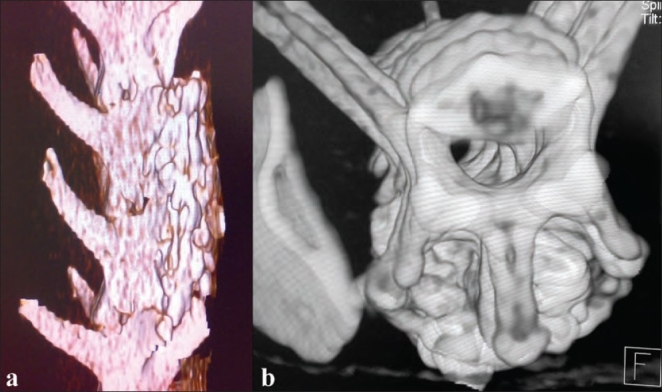
CT scans 3-D reconstructed images showing Grade 3 fusion in Group I at 24 weeks. Continuous bony bridge fusion can be seen over posterior surface of the spine and between the transverse processes

Anteroposterior radiographs of lumbar spines were evaluated by two independent radiologists who were blinded to the groups; Kappa test was used to test the interobserver agreement. Radiographic score of Curylo *et al*.^17^ was used to assess the fusion which is as follows: four points were given when continuous bridging bony mass was present on both sides without lucency; three points, when a continuous bony mass was present bilaterally with lucency on one side; two points, when a bony mass was present on both sides with lucency bilaterally; one point, when bony mass was present on one side only and zero point, when no bridging bony mass was seen. A solid fusion was said when at least three points were scored on radiographs. Fusion was assessed on CT scan using axial view and 3D reconstructed images and graded as follows: Grade 3: continuous intersegmental bony bridge fusion seen; Grade 2: doubtful intersegmental bony bridge fusion seen; Grade 1: no intersegmental bony bridge fusion seen [Figure 1].

#### Manual palpation test

The lumbar spine was excised *en bloc* from all animals. After the division of supraspinous and infraspinous ligaments, the intervertebral disc was excised, thus leaving the graft material as the only tissue connecting the adjacent vertebrae. This was performed at the operated level and, for comparison at the adjacent non-operated levels above and below. The results of the manual palpation test^18^ were classified as solid fusion, when no vertebral movements were detected at the operated level and as uncertain when vertebral movements were present at the operated levels but markedly reduced compared with the adjacent levels. When vertebral movements were equal or slightly reduced when compared with the adjacent levels, the results were classified as pseudoarthrosis.

#### Histological technique

Sections from the surgical site were taken, processed and embedded in paraffin wax. Thin sections of about 3-4 *μ*m were cut and stained with Hematoxylin and Eosin (H and E) stain, and then studied under light microscope for morphological features. These sections were also subjected to immunohistochemistry using anti-osteonectin antibody for the confirmation of osteoblastic activity. They were evaluated by two independent histopathologists who were blinded to the groups.

The osteoblastic activity on H and E stain was graded based on the amount of active osteoblasts seen. It was graded as Grade 1: when there was minimal osteoblastic activity; Grade 2: when there was moderate osteoblastic activity and Grade 3: when significant osteoblastic activity was observed. On immunohistochemistry grading was done from 1-3 depending upon the intensity of stain and number of positive cells.

The new bone formation (woven and lamellar bone) was also evaluated and graded. Grade 1 was given when there were small and patchy areas of new bone formation; Grade 2 was assigned when there was moderate amount of new bone formation and Grade 3, when significant amount of new bone formation was seen.

### Statistical analysis

Data was first analyzed using inter-observer agreement (κ) kappa. Fusion rates among the graft material groups were compared using Fisher’s exact test. A one-way analysis of variance (ANOVA)^19^ test was used to make comparisons between individual groups. Results were considered significant when F > F crit and *P* < 0.05 (F ratio stands for- F= (found variation of the group averages)/(expected variation of the group averages) F crit= F critical value).

## RESULTS

Of the 40 rabbits operated, 37 (92.5%) had no complications, one (2.5%) had superficial infection (which healed with antibiotics), one (2.5%) had wound dehiscence (which was repaired with secondary suturing) and one (2.5%) had peritoneal injury while procuring the graft from iliac crest (which was repaired immediately). All the rabbits withstood the surgery well and there was no mortality.

At eight weeks, none of the groups showed solid fusion on X-rays, CT scans and on manual palpation test. Histologically, Group I, III and IV showed comparable osteoblastic activity on both H and E and immuno-histochemistry when compared statistically [(F = 2.79 < Fcrit = 3.35) and *P* > 0.05]. Group II stimulated minimum osteoblastic activity at the end of eight weeks in comparison to all the other groups. There was also an attempt of lamellar bone formation seen in only Group I.

At 24 weeks inter-observer agreement (kappa) was 0.89. Statistically, on X-ray, Group I showed best results when compared to other groups [(F = 45.66 > Fcrit = 3.23) and *P* < 0.05]. Group III proved to be statistically better than Group IV [(F = 32.66 > Fcrit = 5.31) and *P* < 0.05]. Group II was the weakest with no evidence of fusion on X-rays. Statistically similar results were obtained on CT scans at 24 weeks [Figure 1a and b]. On manual palpation test, Group I showed better results when compared to the other groups. However, on statistical analysis the results of Group I and Group III were comparable to each other [(F = 0.4 < Fcrit = 5.32) and *P* > 0.05]. The interobserver agreement (kappa) was 0.80. All the groups showed increase in the osteoblastic activity as compared to the picture at 8 weeks. Group I showed better osteoblastic activity as compared to other groups; however, statistically, all the groups showed comparable results to each other except group II. Histologically, woven bone formation was seen in all the groups except Group II; the lamellar bone formation was seen only in Group I and Group III. On statistical analysis, amount of lamellar bone formation seen in group I was found to be significantly more than group III. Even at the end of 24 weeks, no new bone formation was seen in Group II. The interobserver agreement (kappa) was 0.92.

**Figure 2:**
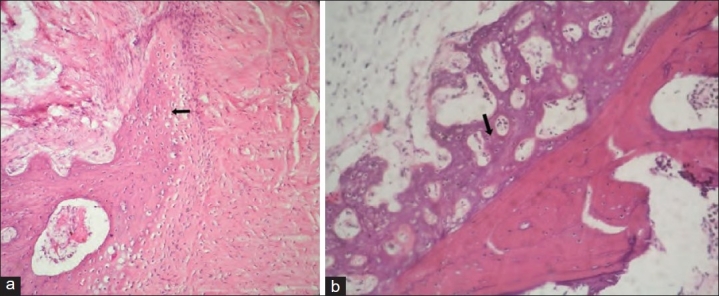
H and E stained section of grafted site in Group I (a) 200× magnification showing new bone formation (woven type). Osteoblasts are present haphazardly dispersed in osteoid tissue (at 8 weeks). (b) 200× magnification showing well incorporated bone graft substitute with evidence of new bone formation (woven type) (at 24 weeks)

**Figure 3:**
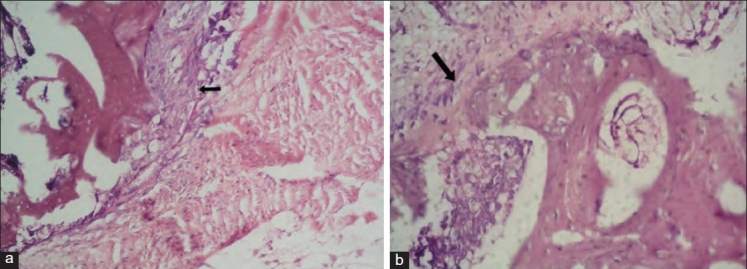
H and E stained section of grafted site in Group III (a) 200× magnification showing moderate osteoblastic activity (at 8 weeks). (b) 200× magnification showing intense osteoblastic activity and small areas of new bone formation (woven type) (at 24 weeks)

**Figure 4:**
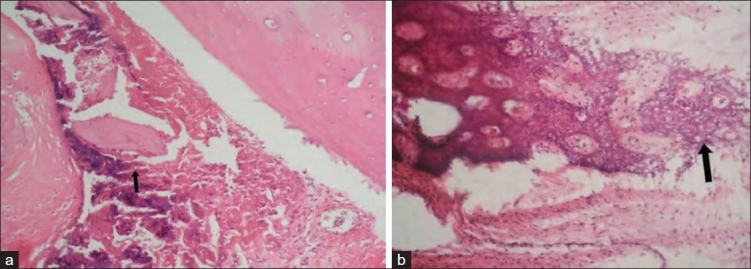
H and E stained section of grafted site in Group IV (a) 200× magnification showing osteoblastic activity around autologous grafts (at 8 weeks). (b) 200× magnification showing moderate osteoblastic activity (at 24 weeks)

The results of X-rays, CT scans, manual palpation test and histology at eight and 24 weeks are shown in Table 1. Histological examples of group I, III and IV at eight and 24 weeks are shown in [Figures 2–4,a,b].

**Table 1:** Results in the respective groups at 8 and 24 weeks

Modalities of fusion assessment	Follow up (weeks)			Groups				
			I β-TCP+BMA	II BMA	III β-TCP	IV ABG
X-ray*	8		0	0	0	0
	24		2 (40)	0	2 (40)	0
CT scan**	8		0	0	0	0
	24		2 (40)	0	1 (20)	0
Manual palpation***	8		0	0	0	0
	24		2 (40)	0	1 (20)	0
Histology				
Osteoblastic activity (Anti-osteonectin stain)	8	Gr. 1	-	1 (20)	3 (60)	3 (60)
		Gr. 2	5 (100)	-	2 (40)	2 (40)
		Gr.3	-	-	-	-
	24	Gr. 1	-	2 (40)	1 (20)	-
		Gr. 2	1 (20)	-	2 (40)	4 (80)
		Gr.3	4 (80)	-	2 (40)	1(20)
New bone formation	8	Woven bone	5 (100)	-	5 (100)	2 (40)
		Lamellar bone	2 (40)	-	-	-
	24	Woven bone	5 (100)	-	5 (100)	4 (80)
		Lamellar bone	5 (100)	-	2 (40)	-

Figures in parentheses are in percentage

* Fusion was considered when atleast Curylo’s Criteria 3 was met on X-rays

** Fusion was considered when Grade 3 type of fusion was seen on CT scans

*** Fusion was considered when no movements were present at the fusion site on manual palpation test, Total no. of rabbits n=40 {10 per each group(five for eight weeks and five for 24 weeks follow-up)}, β-TCP: Beta Tricalcium phosphate, BMA: Bone marrow aspirate, ABG: Autologous bone graft

## DISCUSSION

Schimandle and Boden reviewed the use of animal models to study spinal fusion. The rabbit is one of the smallest animals with lumbar spinal anatomy comparable with human beings and enough iliac crest bone to provide autologous graft for spinal fusion. It is also critical to use skeletally mature animals to determine the fusion rate.^20^ It is obvious from our results that the composite graft consisting of beta tricalcium phosphate and bone marrow aspirate is superior to all the other groups. Bone marrow alone, though osteogenic in nature, requires a carrier to help in inducing osteoblastic activity. Being in a fluid state there is a possibility that bone marrow gets drained or absorbed to other sites from the decorticated fusion site and hence a substantial amount of osteoprogenitor cells are not available for inducing osteoblastic activity. On the other hand when it was combined with a carrier in the form of beta tricalcium phosphate having a porosity of 60% with pore width 100-500 *μ*m, it got embedded in the carrier and provided the osteogenic property to the osteoconductive material and hence proved superior to all the groups. The main attraction of calcium ceramic is that it is bioactive. According to the definition given by Williams, bioactive denotes “a material which induces specific biological activity.” Calcium phosphate ceramics are able to form a strong bond and can be resorbed.^21^–^23^ The tissue response, which we observed with respect to calcium phosphate ceramic, is comparable to that reported by Flatley *et al*.^24^ They observed that at eight weeks after the implantation there was a development of woven bone, which continued to proceed rapidly through scaffold of interconnecting channels. At 24 weeks, they also observed dense contiguous sheaths of mature lamellar bone throughout the depth of the porous ceramic block and extensive osteoblastic activity. The rate of fusion ranging from 50-70% and 0-100% has been reported respectively when autologous bone and alternative graft were used in experimental posterolateral fusion by Cinnotti *et al*.^18^ In our study autologous bone graft gave no evidence of solid fusion on manual palpation and radiologic evaluation at the end of 24 weeks. However, beta tricalcium phosphate when used alone showed evidence of solid fusion in 20% but when used as a composite with bone marrow aspirate, it gave rate of 40%. It appears that since the spines of the rabbits were not fixed by any instrumentation the rate of fusion was low and could have been increased if the spine was fixed. Although the radiographic results may be affected by the non-resorbed ceramic, which may render it more difficult to diagnose the presence of pseudoarthrosis, both plain X-ray and CT scan showed that even when a continuous bony mass was present, it appeared to be more homogenous and ceramic granules were more adherent to the transverse process in rabbits treated with beta tricalcium phosphate and bone marrow aspirate composite than in those with beta tricalcium phosphate. Similar findings were also observed by Cinotti *et al*.^18^ According to the manufacturer’s indications (Synthes), the beta tricalcium phosphate (chronOS) should undergo resorption by six to 18 months. In our study, 80% animals showed degradation above 50%, however, since study was limited to six months it could not provide long term behaviour of the graft material. When we compared our study with similar studies in the literature, as shown in Table 2, it was found that Kai *et al*.^25^ at 12 weeks follow-up and Ori *et al*.^1^ at 24 weeks follow-up had no fusion, though Cinnoti *et al*.^18^ at eight weeks had 30% fusion when they used beta tricalcium phosphate alone as a graft material for spinal fusion. In our study 40% fusion was seen at 24 weeks on X-ray, but on CT scan evaluation, fusion rate in our study came out to be 20%. In our view these authors should have evaluated the fusion with the help of CT scan also, as the shadows obtained on roentgenogram for fusion assessment may be deceptive at times as is obvious from our study (In group III there was fall in fusion rate when the same rabbits were subjected to CT scan). When beta tricalcium phosphate was used as composite graft with bone marrow aspirate, the fusion was 50% in the study by Cinotti *et al*.^18^ and we had a fusion rate of 40%, both on X-ray and CT scan evaluation. Surprisingly, the results of posterolateral spinal fusion with autologous bone graft, which is considered as a gold standard, were found to be significantly variable.^1^,^17^,^18^,^25^–^27^ The autologous bone graft histologically started showing evidences at eight weeks and improved at 24 weeks suggesting that autologous bone graft may need more time to form solid fusion. The method adopted by authors like Cinotti *et al*.^18^ and by us for mechanical testing of the fusion by manual palpation is very subjective. It would have been more reliable if some mechanical device, as used by Motomiya *et al*.^28^ or by Boden *et al*.^26^ would have been used. We also feel that quality and quantity of fusion cannot be decided based on a single criteria or test. The final analysis should be done after taking into consideration all the three parameters, namely, radiological, mechanical and histological. One of the limitations of the study is that no control group was utilized as a “no bone graft group” and no “time-zero” specimens were examined. Assuming that the inherent fusion potential for these animals is high, and a large numbers of osteogenic cells were present at time zero and this would have influenced the results; but then these situations were present in all the groups, as decortication and preparation of the bed for fusion was done in all the groups. To conclude, the composite graft of beta tricalcium phosphate and bone marrow aspirate can be used as an alternative to autologous iliac crest bone graft for postero-lateral spinal fusion. In clinical situations where autologous bone grafts are not available in large quantities such composite grafts would be a boon. Secondly complications associated with the harvesting of autologous bone grafts can be avoided with the use of such composite grafts.

**Table 2:** Comparative analysis of fusion rates on radiology with other studies

Authors	Followup (weeks)	Graft materials used with respective fusion rate			
		β-TCP+BMA%	BMA%	β-TCP%	ABG%
Kai *et al*. (2003)	12	-	-	0	50
Cinotti *et al*.(2004)	8	50	-	30	25
Curylo *et al*.(1999)	12	-	-	-	25
Ori *et al*.(2005)	24	-	-	0	66.7
Baramiki *et al*.(2000)	20	-	-	-	100
Present study					
X-ray	8	0	0	0	0
	24	40	0	40	0
CT scan	8	0	0	0	0
	24	40	0	20	0

## References

[1] Orii H, Sotome S, Chen J, Wang J, Shinomiya K (2005). Beta-tricalcium phosphate (beta-TCP) graft combined with bone marrow stromal cells (MSCs) for postero-lateral spine fusion. J Med Dent Sci.

[2] Fernyhough JC, Schimandle JJ, Weigel MC, Edwards CC, Levine AM (1992). Chronic donor site pain complicating bone graft harvesting from the posterior iliac crest for spinal fusion. Spine.

[3] Kurz LT, Garfin SR, Booth RE (1989). Harvesting autogenous iliac bone grafts. A review of complications and techniques. Spine.

[4] DePalma AF, Rothman RH (1992). The nature of pseudoarthrosis. 1968. Clin Orthop Relat Res.

[5] Steinmann JC, Herkowitz HN (1992). Pseudarthrosis of the spine. Clin Orthop Relat Res.

[6] Jarcho M (1981). Calcium phosphate ceramics as hard tissue prosthetics. Clin Orthop Relat Res.

[7] Natrajan M, Dhanpal R, Kumaravel S, Selvaraj R, Uvaraj NR (2003). The use of bovine calcium hydroxyapatite in filling defects following curettage of benign bone tumours. Indian J Orthop.

[8] Matsumine A, Myoui A, Kusuzaki K, Araki N, Seto M, Yoshikawa H (2004). Calcium hydroxyapatite ceramic implants in bone tumour surgery. A long-term follow-up study. J Bone Joint Surg Br.

[9] Reddy R, Swamy MK (2005). The use of hydroxyapatite as a bone graft substitute in orthopedic conditions. Indian J Orthop.

[10] Spivak JM, Hasharoni A (2001). Use of hydroxyapatite in spine surgery. Eur Spine J.

[11] Boden SD, Schimandle JH (1995). Biologic enhancement of spinal fusion. Spine.

[12] Ohura K, Bohner M, Hardouin P, Lemaître J, Pasquier G, Flautre B (1996). Resorption of, and bone formation from, new beta-tricalcium phosphate-monocalcium phosphate cements: an in vivo study. J Biomed Mater Res.

[13] Kurashina K, Kurita H, Wu Q, Ohtsuka A, Kobayashi H (2002). Ectopic osteogenesis with biphasic ceramics of hydroxyapatite and tricalcium phosphate in rabbits. Biomaterials.

[14] Hulbert SF, Cooke FW, Klawitter JJ, Leonard RB, Sauer BW (1973). Attachment of prosthesis to musculoskeletal system by tissue ingrowth and mechanical inter locking. J Biomed Mater Res.

[15] Fleming JE, Cornell CN, Muschler GF (2000). Bone cells and matrices in orthopedic tissue engineering. Orthop Clin North Am.

[16] Muschler GF, Boehm C, Easley K (1997). Aspiration to obtain osteoblast progenitor cells from human bone marrow: the influence of aspiration volume. J Bone Joint Surg Am.

[17] Curylo LJ, Johnstone B, Petersilge CA, Janicki JA, Yoo JU (1999). Augmentation of spinal arthrodesis with autologous bone marrow in a rabbit postero-lateral spine fusion model. Spine.

[18] Cinotti G, Patti AM, Vulcano A, Della Rocca C, Polveroni G, Giannicola G (2004). Experimental postero-lateral spinal fusion with porous ceramics and mesenchymal stem cells. J Bone Joint Surg Br.

[19] Danial Solan How to compare data sets-ANOVA. http://www.isixsigma.com/library/content/c021111a.asp.

[20] Schimandle JH, Boden SD (1996). Spine update: The use of animal models to study spinal fusion. Spine Update. Spine.

[21] Renooij W, Hoogendoorn HA, Visser WJ, Lentferink RH, Schmitz MG, Van Ieperen H (1985). *In vivo* Bioresorption of ceramic strontium-85-labeled calcium phosphate implants in dog femora. A pilot study to quantitate bioresorption of ceramic implants of hydroxyapatite and tricalcium orthophosphate. Clin Orthop Relat Res.

[22] Passuti N, Daculsi G, Rogez JM, Martin S, Bainvel JV (1989). Macroporous calcium phosphate ceramic performance in human spine fusion. Clin Orthop Relat Res.

[23] LeGeros RZ, Parsons JR, Daculsi G, Driessens F, Lee D, Liu ST (1988). Significance of the porosity and physical chemistry of calcium phosphate ceramics. Biodegradation-bioresorption. Ann N Y Acad Sci.

[24] Flatley TJ, Lynch KL, Benson M (1983). Tissue response to implants of calcium phosphate ceramic in the rabbit spine. Clin Orthop Relat Res.

[25] Kai T, Shao-qing G, Geng-ting D (2003). *In vivo* evaluation of bone marrow stromal-derived osteoblasts-porous calcium phosphate ceramic composites as bone graft substitute for lumbar intervertebral spinal fusion. Spine.

[26] Boden SD, Martin GJ, Morone M, Ugbo JL, Titus L, Hutton WC (1999). The use of coralline hydroxyapatite with bone marrow, autogenous bone graft, or osteoinductive bone protein extract for postero-lateral lumbar spine fusion. Spine.

[27] Baramki HG, Steffen T, Lander P, Chang M, Marchesi D (2000). The efficacy of interconnected porous hydroxyapatite in achieving postero-lateral lumbar fusion in sheep. Spine.

[28] Motomiya M, Ito M, Takahata M, Kadoya K, Irie K, Abumi K (2007). Effect of Hydroxyapatite porous characteristics on healing outcomes in rabbit postero-lateral spinal fusion model. Eur Spine J.

